# The prevention and eradication of smallpox: a commentary on Sloane (1755) ‘An account of inoculation’

**DOI:** 10.1098/rstb.2014.0378

**Published:** 2015-04-19

**Authors:** Robin A. Weiss, José Esparza

**Affiliations:** 1 Division of Infection and Immunity, University College London, London WC1E 6BT, UK; 2 Institute of Human Virology, University of Maryland School of Medicine, Baltimore, MD 21201, USA

**Keywords:** smallpox, inoculation, variolation, vaccination

## Abstract

Sir Hans Sloane's account of inoculation as a means to protect against smallpox followed several earlier articles published in *Philosophical Transactions* on this procedure. Inoculation (also called ‘variolation’) involved the introduction of small amounts of infectious material from smallpox vesicles into the skin of healthy subjects, with the goal of inducing mild symptoms that would result in protection against the more severe naturally acquired disease. It began to be practised in England in 1721 thanks to the efforts of Lady Mary Wortley Montagu who influenced Sloane to promote its use, including the inoculation of the royal family's children. When Edward Jenner's inoculation with the cow pox (‘vaccination’) followed 75 years later as a safer yet equally effective procedure, the scene was set for the eventual control of smallpox epidemics culminating in the worldwide eradication of smallpox in 1977, officially proclaimed by WHO in 1980. Here, we discuss the significance of variolation and vaccination with respect to scientific, public health and ethical controversies concerning these ‘weapons of mass protection’. This commentary was written to celebrate the 350th anniversary of the journal *Philosophical Transactions of the Royal Society*.

## 1. Introduction

Immunization against infectious diseases has protected more children and adults from untimely deaths than any other form of treatment, and smallpox was the first illness to be prevented in this way. When Sir Hans Sloane gave his account of inoculation in 1736 [1], the concepts of contagion and immunity to re-infection were already well understood, although it would take another 150 years before the germ theory of infectious disease led to the identification of specific pathogens by Louis Pasteur and Robert Koch. Reports in the *Philosophical Transactions* played a leading role in establishing the efficacy and relative safety of inoculation against smallpox.

## 2. Sir Hans Sloane

**Figure 1.**
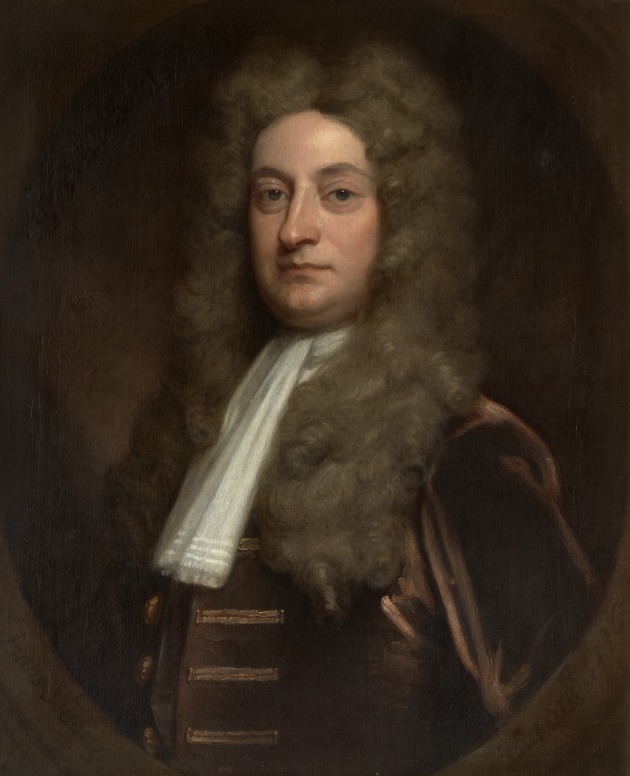
Sir Hans Sloane. Portrait by Godfrey Kneller, 1716. Copyright © The Royal Society.

Sir Hans Sloane (1660–1753) (figure 1) was a polymath with an extraordinary range of interests even for a man of the Enlightenment [2]. He became personal physician to the families of three British monarchs, Queen Anne, George I and George II, a man of public affairs and a philanthropist. Born into an Irish Protestant family in the year that the Royal Society was founded, Sloane studied medicine and took a great interest in natural history, being elected a Fellow of the Royal Society at the age of 25. Although he had already established a medical practice in London, he leapt at the opportunity to accompany the Duke of Albemarle as family physician when Albemarle was appointed Governor of Jamaica in 1687. Sloane collected numerous botanical specimens and kept a detailed account of his observations on the natural history and people of the Caribbean islands during his 15 months' sojourn there, which he later published in two volumes of exemplary natural history. He disliked the bitter cocoa beans of the New World and advocated the use of a concoction of cocoa, sugar and milk, which we know as chocolate, mostly as a medicinal beverage. In 1719, he became President of the Royal Society of Physicians London. He was also appointed physician-general to the army in 1722. He served as Secretary to the Royal Society (1693–1713) when he had editorial responsibility for *Philosophical Transactions* [3], and in 1727, he succeeded Sir Isaac Newton as President of the Society, stepping down from that post 13 years later at the age of 80.

Today, Sloane is best remembered as the founder of the British Museum. He was an avid collector of human artefacts as well as natural history. He left his personal collections to the Crown on the provision that they would be well housed and curated for the benefit of the public, and that his daughters would receive a stipend. Parliament eventually approved this bequest and Sloane's collections became the basis of the British Museum in Bloomsbury and its offshoot, the Natural History Museum in South Kensington. He donated the land on which the Physick Garden stands in Chelsea to the Apothecaries' Society, which still exists as an eighteenth century medical herbarium. The influence of Sir Hans Sloane on this area of London, where he bought the manor of Chelsea, lasts to this day in place names such as Sloane Square and Hans Crescent. As a benefactor, Sloane was a founding supporter of the Foundling Hospital for Children in Bloomsbury, which was established by three notable friends, the merchant seaman Thomas Coram, the composer George Frideric Handel and the artist William Hogarth.

For a man with such an enquiring mind, Sir Hans published relatively few primary research papers or treatises. Perhaps this was due to his multiple offices and duties. His paper on smallpox describes how he became acquainted in 1721 with the benefits of intradermal inoculation of a small dose of smallpox-infected fluid from a vesicle, and became an advocate of the procedure among the royal family and the medical profession. This paper is just what its title suggests: it is an account or reminiscence of Sloane's introduction of the practice of inoculation and does not really represent a remarkable discovery. Nonetheless, it is full of insights into the disease and an important means to prevention.

Sloane states that his ‘Account of inoculation’ was ‘given to Mr Ranby, to be published, anno 1736’. In fact, it was not published until 1755, just over a year after Sloane's death. Why Sloane provided this account at this time to John Ranby FRS remains obscure as Ranby was not involved in editing *Philosophical Transactions*. By 1755 his ‘co-author’ (the person who communicated the paper to the Royal Society), the Reverend Dr Thomas Birch, was the Secretary of the Society with responsibility for *Philosophical Transactions*. Birch would have had access to Sloane's papers as he was a founding trustee of the British Museum where the papers were (and are) stored. Sloane's collections of books, antiquities, natural curiosities and manuscripts formed the museum's original collection, including material on Sloane's activities as Secretary and later President of the Royal Society. The reason for the long delay in publication is not clear, but it seems possible that when Birch was sorting through Sloane's papers, he came across this piece and thought that it should finally see light of day.

**Figure 2.**
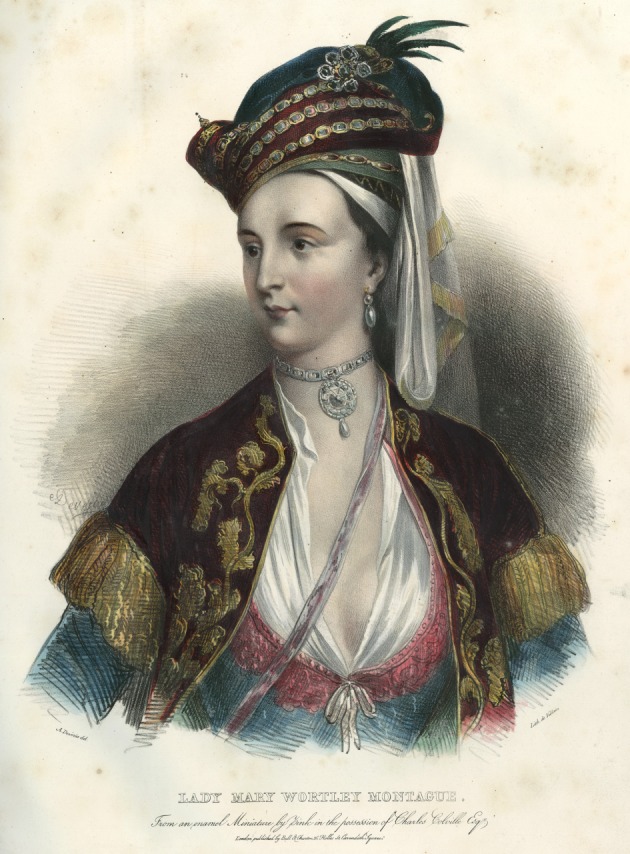
Lady Mary Wortley Montagu in Turkish costume. Copyright © National Portrait Gallery, London.

Sloane's paper acknowledges that the successful promulgation of inoculation owed much to two remarkable women. The first was Lady Mary Wortley Montagu (1689–1762) (figure 2) who first observed variolation in Turkey and who had her son inoculated there in 1717 and her daughter in England during the 1721 epidemic (box 1). The second was Princess Caroline (1683–1737), wife of the future George II, who sought Sloane's advice on whether her own children should be inoculated. Sloane relates that her decision to take this risk followed two successful trials of variolation: the first was on adults in Newgate prison under death sentence who ‘volunteered’ to be variolated in exchange for their release if they survived, one of whom was sent to have contact with a smallpox patient as a test of protection; the second was on ‘charity children’, orphans in the parish of St James in London. The ethics of enrolment into clinical trials may have changed since those days but the concept of step by step inquiry from safety to efficacy remains unchanged.

Box 1. **Lady Mary Wortley Montagu (1689–1762) and smallpox, the ‘Destroying Angel’.**

Lady Mary Wortley Montagu, daughter of the Marquess of Dorchester, was a highly educated and independent minded person who mixed with literary and scientifically minded people and who kept a salon. In 1712, she escaped from an arranged marriage intended by her father by eloping with and marrying Sir Edward Wortley Montagu. In 1716, she went to live for two years in Turkey when her husband was appointed as British ambassador to the Ottoman court in Constantinople. She learned Turkish and Greek, visited the women in their segregated quarters, made friends and learned about Turkish customs. She wrote many ‘Letters from the Embassy’ (an eighteenth century form of blog) to her friends at home about her experiences [4]. In one written in April 1717, she describes a visit to Sofia, which was then part of the Ottoman Empire, where she went to the hammam (public baths):

I am now got into a whole new world. It is hard to tell the mistresses from their servants, for they were all in the state of nature, that is, in plain English, stark naked; some working, others drinking coffee or sherbet. In short, ‘tis the women's coffee house, where all the news of the town is told, scandal invented, &c.

Lady Mary's brother had died of smallpox and when she herself suffered from the disease in 1715 she developed severe pock marks on her face and regretted the loss of her famed beauty. While in Turkey, she became interested in smallpox prevention by variolation. In another letter also written in April 1717, she tells of the inoculation against smallpox:

A propos of distempers, the small-pox, so fatal, and so general amongst us, is here entirely harmless, by the invention of engrafting, which is the term they give it. There is a set of old women, who make it their business to perform the operation, every autumn, in the month of September, when the great heat is abated. People send to one another to know if any of their family has a mind to have the small-pox; they make parties for this purpose, and when they are met (commonly fifteen or sixteen together) the old woman comes with a nut-shell full of the matter of the best sort of small-pox, and asks what vein you please to have opened. She immediately rips open that you offer to her, with a large needle (which gives you no more pain than a common scratch) and puts into the vein as much matter as can lie upon the head of her needle, and after that, binds up the little wound with a hollow bit of shell, and in this manner opens four or five veins.

Lady Mary had her own son inoculated, witnessed by the British doctor at the embassy, Charles Maitland. She wrote:

The boy was engrafted last Tuesday, and is at this time singing and playing and very impatient for his supper … I am patriot enough to take the pains to bring this useful invention into fashion in England, and I should not fail to write to some of our doctors very particularly about it, if I knew any one of them that I thought had virtue enough to destroy such a considerable branch of their revenue, for the good of mankind*.*

Back in England, Lady Mary indeed encountered resistance to this ‘dangerous Oriental method’. But in Sloane and Maitland, she found like minds committed to disease prevention. Early in 1721, it was so warm that roses bloomed in January and smallpox ‘went forth like a Destroying Angel’. Lady Mary called upon Charles Maitland to inoculate her three year old daughter but he hesitated as it was one thing to follow the custom in Turkey, but another to do it in London. He made sure he had two witnesses from the Royal College of Physicians before performing the operation. One was James Keith, a friend of Maitland who had lost two sons to smallpox in 1717, and the other (though not recorded) may well have been the President, Sir Hans Sloane.

Sloane writes that he felt unable to recommend firmly to Princess Caroline that her children should be variolated but that on her further questioning, he stated that the consequence of not undergoing the procedure might be much worse. This was a very diplomatic reply so that the Princess had to make the decision herself. She then urged Sloane to speak to the children's grandfather, George I, who condoned it (even though he was barely on speaking terms with the children's father, the Prince of Wales). Inoculating the royal children without ill effect helped to make variolation widely acceptable among the English aristocracy and gentry. The procedure was also introduced in New England [5] (see box 5). In France, however, it was thought to be reckless [6] and Voltaire commented on the difference in attitudes between the two nations with characteristic acuity (box 2).

Box 2. **Voltaire and the different perceptions of variolation in England and France.**

Voltaire spent nearly three years in exile in London in 1726–1728. He relished the greater freedom of speech than that allowed in Paris under Louis XV's absolute monarchy, and he probably attended soirées at the Royal Society. In the English translation of his *Philosophical Letters* [6] published in 1734 he comments with typical wit:

It is inadvertently affirmed in the Christian countries of Europe that the English are fools and madmen. Fools, because they give their children the small-pox to prevent their catching it; and madmen, because they wantonly communicate a certain and dreadful distemper to their children, merely to prevent an uncertain evil. The English, on the other side, call the rest of the Europeans cowardly and unnatural. Cowardly, because they are afraid of putting their children to a little pain; unnatural, because they expose them to die one time or other of the small-pox. But that the reader may be able to judge whether the English or those who differ from them in opinion are in the right, here follows the history of the famed inoculation, which is mentioned with so much dread in France.

Voltaire went on to describe the procedure and its low relative risk compared to smallpox itself, possibly relying on James Jurin's calculations published in *Philosophical Transactions*. It is interesting that some 30 years later, in 1760, the Dutch/Swiss mathematician Daniel Bernoulli read to the French Royal Academy of Sciences his famous mathematical model paper [7] describing how the control of smallpox epidemics by the large scale use of variolation would result in benefits not only to the individuals, but also to the state and society at large, which today is a key concept of many public health interventions. Bernoulli himself cited a paper published in *Philosophical Transactions* in 1693 by Edmund Halley who compiled the first life expectancy tables.

Had Bernouilli's recommendations been implemented, the death of Louis XV could have been avoided. In 1774, at the age of 64, Louis XV died of smallpox in the palace of Versailles. According to a witness, ‘The air of the palace was infected; more than fifty persons took the smallpox, in consequence of having merely loitered in the galleries of Versailles, and ten died of it.’ With the death of Louis XV, his grandson became Louis XVI and his young wife, Marie Antoinette, became the queen of France. Marie Antoinette was immune to smallpox because as a child in Vienna she had already suffered from a mild form of the disease. In 1767 her mother, the Empress Marie Theresa of Austria, promoted variolation in Austria after recovering from severe smallpox. Following the example of her mother, Marie Antoinette convinced the new king and his brothers to be variolated, perhaps providing further evidence that royal initiatives can go a long way in adopting scientific recommendations. In modern times, when Princess Diana was photographed hugging a man with AIDS, it showed the world that AIDS patients need not be regarded as ‘lepers'.

Ironically, when Edward Jenner developed vaccination, it was taken up much more quickly and widely in post-revolution France than in England [8]. While there is no doubt that the success of variolation made the adoption of the concept of vaccination easier, the English variolators opposed the new vaccinators because they did not want to change a lucrative practice. In France, however, the practice of variolation was less well established even 60 years after Voltaire's letter, yet the terror invoked by smallpox eclipsed that of the guillotine. The advantage of the safer vaccination was soon perceived as an efficacious protection without the risk of untoward effects.

## 3. Smallpox

**Figure 3.**
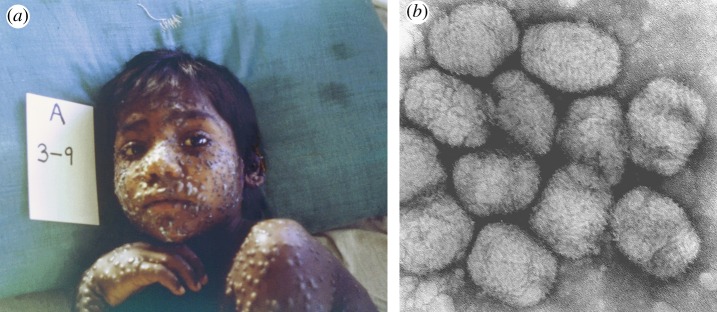
(*a*) Child with smallpox; (*b*) Variola virus particles. (Images from the CDC Public Health Image Library).

Smallpox (figure 3*a*) is a relatively recent disease of humankind [9,10]. Like many epidemic infectious diseases, the causative agent, variola virus (figure 3*b*), probably crossed over from an animal source after humans attained sufficient population density to sustain the propagation and diversification of the variola virus independently from its original host. It was not until the 1890s that filterable viruses were first distinguished from bacteria that were retained by porous filters. Orthopox viruses, to which variola and vaccinia belong, were first identified as filterable agents by Adelchi Negri's study of vaccinia in 1906 [11]. The story of the rise and fall of smallpox is told by Frank Fenner and the pioneers of eradication [11] as well as several more recent popular books [12–15].

Depending of the severity of clinical disease, smallpox viruses used to be divided between the most virulent form, *Variola major*, with a mean case fatality rate of 25–30%, and a milder form, *Variola minor*, with a fatality rate of approximately 15%, but this phenotypic classification only partially correlates with phylogenetic comparisons of virus strains [16]*.* Many of those who survived showed permanent signs of past infection, such as blindness and pock marks on the skin, but they acquired long-term immunity that rendered them resistant to re-infection. The lack of an animal reservoir, combined with the appearance of obvious signs of infection in all cases, and lack of virus persistence in recovered individuals, permitted the containment and eventual eradication of smallpox as a naturally occurring disease.

Although it is thought that the pock marks on the cheeks of the mummified Egyptian Pharaoh Ramses V (approx. 1200 BCE) might represent smallpox, it has not been possible to isolate variola virus DNA from ancient mummified material [17]. The pocks are difficult to distinguish from chickenpox caused by varicella-zoster virus, a type of herpesvirus infection which has co-evolved with hominoids ever since we diverged from the great apes. There are no records of a smallpox-like disease in the Bible or Greco-Roman writings [16]. The first reliable description of smallpox was made in China and dates from the fifth century of the Christian Era (CE) although it may have been present in Asia earlier. The first recorded description of smallpox in Western Europe was made in 581 by the Bishop Gregory of Tours. The Persian physician Abu Bakr Muhammad ibn Zakariyya Al-Razi (854–925 CE) [18] wrote a monograph on smallpox and measles (*Kitab al-Jadari wa'l-hasba*) which is the oldest medical treatise on these two diseases as distinct ailments (interestingly, he considered measles to be the more virulent infection). Al-Razi advocated a way to prevent smallpox affecting the cornea and thereby causing blindness [18]. He wrote: ‘As soon as the symptoms of smallpox appear, drop rose water into the eyes from time to time, for if the disease be favourable and the pustules few in number, you find that this method of treatment prevents their breaking out in the eyes' (p. 148).

The proximate animal precursor to variola virus remains uncertain. Phylogenetic analysis of the DNA genome of the virus reveals a close similarity to camelpox virus [19] but also to a poxvirus isolated from the West African rodent, the *Tatera* gerbil [16,20]. Using forensic DNA and phylogenetic methods, it is often possible to ascertain which host was the original reservoir by determining whether the genetic variability of one species' microbe is nested within a broader diversity of the other's. Thus, it is clear that the pandemic strain of HIV-1 came from chimpanzees in Cameroon [21], whereas for the tubercle bacillus, human *Mycobacterium tuberculosis* appears to be ancestral to animal strains such as *Mycobacterium bovis* [22]. While the ancestral orthopox virus for *Variola* appears to reside in burrowing rodents, insufficient samples of pox virus isolates have been analysed to discern whether both smallpox and camelpox were independently derived from the rodentpox virus, or smallpox from camelpox, or camelpox from smallpox. If camelpox was the immediate precursor to smallpox, this situation has parallels to the current outbreak in humans of the coronavirus causing Middle East respiratory syndrome (MERS). Serological and genome studies indicate that a virus closely related to MERS is widespread among domestic dromedaries; fortunately, human cases thus far represent either a primary zoonosis or nosocomial infections directly related to the zoonosis [23].

Since the severe epidemic form of smallpox (*Variola major*) was first recorded in East Asia, it is likely to have originated there around 1700 years ago. It is curious that no further field investigations of rodent variola-like viruses have been made since the African gerbil virus was isolated 40 years ago. We therefore propose that it would be informative to characterize pox viruses of gerbil and marmot species indigenous to Mongolia, the Gobi desert and the central Asian steppe. These are the same species, incidentally, that gave rise to the Black Death clades of the plague bacillus *Yersinia pestis* in the fourteenth century; indeed, *Variola major* may have migrated westwards along the same silk route 1000 years earlier. We further propose that several independent cross-species introductions of smallpox may have occurred, as seen for HIV in the twentieth century [21]. Thus, the two major clades of *Variola minor* [16,20] may have separate African origins, and *Variola major* an Asian one.

The virulence of infections can change greatly upon cross-species infection as is well recognized for another pox virus, myxoma virus; it causes mild symptoms in its original reservoir species, the South American cotton-tail rabbit, but became a highly lethal, epizootic infection in the European rabbit, both in Europe and Australia [24]. In evolutionary terms, smallpox can be considered to be a recent human infection which remains highly virulent.

Whatever be its origin, *Variola major* became an exclusively human virus, unlike the cowpox group of viruses which has a broad host range. Smallpox emerged as a major scourge of humankind, spreading all over the inhabited world. As late as 1967, when the World Health Organization (WHO) initiated the Intensified Smallpox Eradication Campaign, the annual number of smallpox cases was estimated to be between 10 and 16 million, with 2.6 million deaths across 31 countries [11]. During the past 500 years, the impact of smallpox was particularly severe when it reached naive populations. For instance, pre-Columbian New World peoples had never experienced smallpox because their ancestors crossed the Bering Strait before it emerged in humans. The high mortality observed in the first American epidemics, authentic examples of ‘virgin soil epidemics’, could have been exacerbated by the genetic constitution of the affected population which limited their capacity to establish appropriate immunological responses to the newly introduced pathogens [25]. Hernan Cortes and his band of adventurers would not have succeeded in overcoming the mighty Aztec Empire in 1521 without the epidemic of smallpox which they unwittingly released into the capital city, Tenochtitlan, the previous year, as told in the chronicles of the conquistador Bernal Diaz del Castillo [26]. Without the devastating effect of smallpox [27] and other viral diseases such as measles, there would not have been an estimated 85–90% fall in the population of indigenous Americans during the sixteenth and seventeenth centuries [28], and therefore little impetus to establish the trans-Atlantic slave trade [9].

The lesson of smallpox in the Spanish colonization of Mexico was not lost on Francisco Pizzaro during his coup against the Incas a few years later. Moreover, in eighteenth century North America, the redcoat Colonel Henry Bouquet sent his commander, General Lord Jeffrey Amherst, a request ‘to inocculate the Indians' by sending smallpox-impregnated blankets to the Native Americans, who, under the leadership of Chief Pontiac, were besieging Fort Pitt (Pittsburgh). In a postscript to his reply on 16 July 1763, Amherst approved this request and added that Bouquet should also ‘try every other method that can serve to extirpate this execrable race’. Continuing concern over the possibility of utilizing smallpox for germ warfare influences the current debate whether to destroy the remaining stocks of the virus officially stored in the former Soviet Union and USA after its eradication as a naturally occurring infection. In 1996, on the 200th anniversary of Jenner's experiment into vaccination, the WHO voted to destroy variola, but this deliberate extinction of a species has yet to be carried out and may be indefinitely delayed [29].

## 4. Variolation

The protection against smallpox by administration of small doses of infected material was called engrafting, inoculation or variolation (*varus* is Latin for pimple). Different forms of variolation had been used for centuries in China and the practice also became widespread throughout the Ottoman Empire and the Arab world [30]. *Philosophical Transactions* published several articles on variolation around the time of Lady Mary Wortley Montagu's campaign. A paper in Latin by Emanuele Timoni FRS was read to the Royal Society in January 1721 about the procedure which he witnessed during the 1714 smallpox outbreak in Constantinople, and Jacob Pylarini discussed the 1716 outbreak in Smyrna (Izmir) as cited by Sloane.

Noah Moxham, the archivist studying *Philosophical Transactions*, has written an interesting blog [31] on how James Jurin, the editor during the 1720s, promoted investigation of variolation in the journal. Jurin's own 1722 paper [32] was a particularly important contribution because he included statistical estimates and tables on the respective mortality rates for inoculation versus natural smallpox infection. Voltaire may have based his commentary on Jurin's paper (box 2).

Another fascinating account in *Philosophical Transactions* [33] translated from Arabic comes from His Excellency the Ambassador to England from Tripoli, Cassem Aga FRS, on how he and his seven siblings were inoculated as children. He adds: ‘The practice is so innocent and so sure that out of an hundred persons inoculated not two die, whereas on the contrary out of an hundred persons that are infected in the natural way there die commonly about thirty. It is withal so ancient in the kingdoms of Tripoly, Tunis and Algier, that nobody remembers its first rise, and it is practiced not only by the inhabitants of the towns, but also by the wild Arabs' [nomads]. Inoculation was also practised in sub-Saharan Africa at this time (see box 5).

Using live pathogenic virus for variolation certainly did carry a risk, and about 2% of those inoculated developed severe smallpox and died (but with refinement of the practice the risk was reduced to around 0.3%). Some newspapers exaggerated accounts of deaths from inoculation. However, in one household, six servants contracted smallpox not long after a child had been inoculated; whether it had spread from the inoculum (which was a real possibility) or was actually a result of natural infection is a moot point since the inoculation took place in the face of an epidemic. Therefore, it was recommended that inoculated individuals should undergo a preparation procedure and be isolated during the vesicular period to avoid starting an outbreak among contacts. Later, vaccination removed these risks, but variolation was preferable to catching natural smallpox, with its much higher mortality and survivors often left pock-marked or blind. However, many physicians resisted the new technique, and some clergymen declared that taking measures to prevent smallpox was acting against God's will.

Sloane [1] also considered familial susceptibility to severe disease in mentioning the one nearly fatal consequence of variolation under his care, that of the son of the Duke of Bridgewater. He noted that many members of this family had died of natural smallpox before the Duke's son was variolated, but that his daughter who received a similar dose of the same incoculum survived without ill effect.

## 5. Vaccination

The greatest advance in the prevention of smallpox was surely Edward Jenner's demonstration that inoculation of cowpox protected against smallpox [34]. He called the procedure vaccine inoculation (‘vacca’, is Latin for cow); Richard Dunning, a surgeon in Plymouth, coined the term vaccination in 1803 [35]. Cowpox can cause a mild disease in humans, who soon recover.

After documenting a number of cases in which previous natural infection by cowpox protected against smallpox (or against successful variolation), on 14 May 1796, Jenner proceeded to vaccinate an eight year old boy named James Phipps. Six weeks later, variolation of the child was attempted without any evidence of infection, providing the first experimental evidence that cowpox elicits immunity to smallpox. At that point, Jenner prepared a communication for the Royal Society, but it was not accepted for publication (box 3). Jenner had to wait two years for new cases of cowpox, to conduct additional experimental inoculations. However, Jenner did not want to risk a new rejection from the Royal Society and privately published his observations in his famous ‘Inquiry’ [34].

Box 3. **Edward Jenner and the Royal Society.**

When we were invited to contribute this article commemorating Sir Hans Sloane's paper on inoculation, our first question was why we had not been invited to feature Edward Jenner's discovery of vaccination instead, given that it has had a more important and long-lasting impact. The answer from the helpful librarians at the Royal Society was that Jenner's paper never passed peer review for *Philosophical Transactions* and was rejected!

Jenner had submitted his initial research on vaccination for publication in 1796 but Sir Joseph Banks, the President of the Royal Society personally declined to accept the paper after taking advice from two reviewers, Lord Somerville, President of the Board of Agriculture, and Sir Everard Home FRS, an eminent London physician. Jenner's biographer, Richard B. Fisher [35], suggests that Banks's rejection of the paper had to do with the ‘paucity of experimental proof in the original paper’, although Somerville pointed him to another country physician, a Dr Dolland, who confirmed Jenner's thesis about the efficacy of vaccination. It seems to have been Banks's decision that influenced Jenner to publish his findings privately.

Incidentally, Jenner had previously withdrawn his paper on the behaviour of the cuckoo which the Committee of Papers (the ‘editorial board’ of *Philosophical Transactions*) had approved for publication, because he had found new evidence that contradicted part of his original manuscript, so it was eventually published a year later [36]. Perhaps Banks thought that Jenner had a propensity to submit work for publication prematurely. But once his research on cuckoos was published it was well received and led to Jenner's election as a Fellow of the Society. In his biographical memoir, Jenner's nephew George Jenner expressed regret that Edward Jenner's work on vaccination had distracted him from his ‘true’ scientific research on cuckoos and avian migration [37]. Both uncle and nephew would surely be pleased that research on the behaviour of cuckoos continues to this day [38].

It might be an interesting addition to the history of science to publish an anthology of ground-breaking discoveries that initially were declined by leading peer-reviewed journals—as well as papers that were published but should have been rejected, such as the false link between autism and MMR vaccination.

Although the protective effective of natural cowpox infection among milkmaids appears to have been known, Jenner was the first to conduct the clinical investigations to provide scientific evidence for unproven folk knowledge (box 4). But the genius of Jenner was not only to provide experimental evidence of the efficacy of vaccination, but also to envisage that this new procedure would eventually eradicate smallpox. As early as 1801 he predicted that ‘The annihilation of the Small Pox, the most dreadful scourge of the human species, must be the final result of this practice’ [41].

Box 4. **Edward Jenner and folk lore.**

‘Where are you going, my pretty maid?’

‘I'm going a-milking, sir’ she said.

‘May I go with you, my pretty maid?’

‘You're kindly welcome, sir’ she said.

‘What is your father, my pretty maid?’

‘My father's a farmer, sir’ she said.

‘What is your fortune, my pretty maid?’

‘My face is my fortune, sir’ she said.

‘Then I can't marry you, my pretty maid.’

‘Nobody asked you, sir’ she said.

Opie and Opie [34] trace early versions of this rhyme back to the troubadours of the fourteenth century with its feminist punch line ‘Nobody asked you, sir’. However, it is not clear whether the line that we consider to be the most telling, ‘My face is my fortune’ predates Jenner's vaccine. It is usually interpreted to mean that as she is poor she depends on being pretty to marry; we propose it reveals traditional folk knowledge that, being a milkmaid, her face will continue to be immune from ugly pock marks and she will not succumb to smallpox.

In his treatise [39], Jenner does not refer directly to folklore of milkmaids being protected from smallpox by previous exposure to cowpox but, rather, he comments on the converse*:* ‘It is a fact so well known among our Dairy Farmers, that those who have the Small Pox either escape the Cow Pox or are disposed to have it slightly’. However, he did mention his own observation, before he experimentally vaccinated James Phipps, that those who had acquired cowpox naturally were immune to variolation and to smallpox. Moreover, awareness of the protective effect of cowpox appears to have been widely appreciated in the west of England, where 22 years before Jenner, Benjamin Jesty in Dorset had inoculated his wife and two sons with cowpox, although he did not conduct a scientific study or follow it up with challenge by variolation.

Jenner's study of the behaviour of cuckoos also seems to be based on commonly believed tales of nature which Jenner patiently tested. Cuckoos could not be heard calling before mid-April and Jenner correctly surmised that they migrated to warmer climates during the winter. Moreover, the cuckoo chick ejecting its non-genetic ‘siblings' from its foster nest must have been noted by observant country folk before Jenner so carefully documented this form of parasitism.

As a physician in rural Gloucestershire, Jenner had the intuition to heed folklore but to test the evidence scientifically. In an accompanying article in this 350th Anniversary issue of *Philosophical Transactions,* John Wood [40] mentions the folk knowledge of the therapeutic power of willow (sallow) infusions long before the scientific discovery of salicylic acid (aspirin). Similarly, the use of chinchona bark for malaria preceded the isolation of quinine.

Vaccination became rapidly adopted worldwide (see box 5 on its early uptake in Massachusetts), and it soon became mandatory in many countries. In the second half of the twentieth century, with the roll-out of vaccination coverage to resource-poor countries, smallpox eventually became confined to two regions of the world, the Horn of Africa and the India–Bangladesh border. As mentioned before, the WHO initiated its Intensified Smallpox Eradication Campaign in 1967 under the inspired leadership of D. A. Henderson, and the last case of naturally occurring smallpox was recorded in Somalia only 10 years later [11,13].

Box 5. **Variolation and vaccination in New England.**

Massachusetts was the first colony of what is today the USA to introduce smallpox inoculation [5] and also the first to introduce vaccination. The Puritan minister Cotton Mather FRS first heard of the procedure in 1706 from his slave, Onesimus, who told him that he had been inoculated as a child in Africa. During the 1721 smallpox epidemic in Boston, and after reading the *Philosophical Transactions* reports from Timoni and Pylarini, Mather convinced Dr. Zabdiel Boylston FRS to initiate variolation in the city, a procedure that was initially conducted amid much controversy. Vaccination was introduced in Boston as early as 1800 by Dr Benjamin Waterhouse (1754–1846), the founding Professor of Medicine in the new medical school at Harvard University. He had received the vaccine from England on threads soaked in cowpox lymph. After vaccinating his children, he challenged them by inoculation with smallpox and showed them to be immune [42]. He went on to put much effort into encouraging public vaccination.

The town of Milton, now part of the Greater Boston, was the first to act in a corporative capacity to extend the benefits of vaccination to all its citizens. In 1809, 337 persons of different ages and conditions were vaccinated, and 12 of them were afterward tested for inoculation of the smallpox and found to be fully protected. The town published a pamphlet describing its experience, entitled: ‘A collection of papers relative to the transactions of the town of Milton, in the State of Massachusetts, to promote a general inoculation of the cow pox, or kine pox, as never failing preventive against small pox infection’.

**Figure 4.**
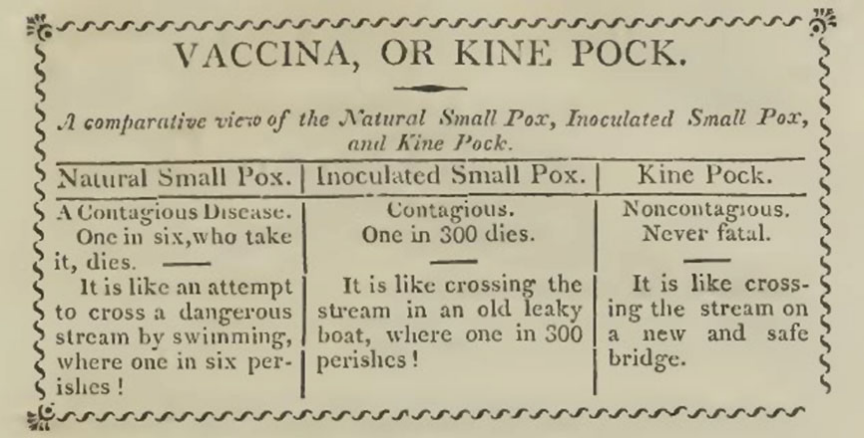
Table in Benjamin Waterhouse's 1809 tract on protection against smallpox in Milton, MA [42].

Consulted by the authorities of Milton in 1809 about the efficacy of vaccination, Waterhouse indicated that he ‘never had the least reason to doubt, but that the Kine Pox [kine is an archaic term for cattle] effectually and forever secures a person from Small Pox’. Waterhouse included in his correspondence to the Committee of the town of Milton, a table (published in the pamphlet mentioned above) which compared the risk of contracting smallpox with the benefits of being variolated or vaccinated. Since this information was addressed to the general public, Waterhouse compared those risks with the more easily recognized experience of crossing a dangerous stream (figure 4).

The real nature of the smallpox vaccine, which we call vaccinia virus, remains a puzzle to this day. For many years it was assumed to be cowpox, but in the mid-twentieth century it became clear that vaccinia stocks were biologically different from cowpox virus [43]. In fact, both cowpox and horsepox were used during the nineteenth century to vaccinate (or ‘equinate’!) against smallpox, and some strains of vaccinia may be derived from horsepox, or from a recombinant with horsepox genes [44,45]. Jenner himself speculated that ‘the preservative of smallpox’ (as he called the vaccine) may have derived from a disease of horses, as modified by its passage through the cow [34]. There also appear to be multiple origins of ‘cowpox’ [46], which probably have reservoirs in rodents that sporadically infect other mammals including, in Jenner's time, cows.

In the early days, preserving vaccine stocks was a problem and finding new cases of cowpox became increasingly rare. Vaccinia started to be maintained by sequential inoculation of children from arm to arm. However, the virus often disappeared or lost its potency. After 1864, retro-vaccination of calves provided a more reliable source of vaccinia, a practice that was initiated in Naples and extended around the world.

There remains concern that smallpox could be revived by rescue of virus from remains of victims preserved in coffins or in the permafrost [17,47], or by malicious means from unrecorded virus stocks, although rapid vaccination should be able to contain an outbreak. Vaccination itself can cause serious disease in immunocompromised people. Therefore, ongoing research is seeking to further attenuate the current ‘modified vaccinia Ankara (MVA)’ while maintaining its immunogenic properties. Furthermore, MVA is also being exploited as a live vector to deliver genes for immunogens of other infectious diseases [48].

## 6. How smallpox prevention led the way for immunization against other diseases

With the development of the germ theory of disease in the second half of the nineteenth century, a large number of specific microorganisms were identified in the aetiology of different infectious diseases, and attempts were made to develop immunization against those diseases. In 1881, Louis Pasteur presented his work on the use of attenuated microorganisms to protect against two animal diseases (chicken cholera and anthrax) at the 7th International Congress of Medicine in London. In that meeting, and to honour Jenner, Pasteur proposed to generalize the term ‘vaccination’ to all protective immunization procedures. In 1885, almost 90 years after the development of vaccination against smallpox, Louis Pasteur and Émile Roux described the second human vaccine, against rabies, although this was an inactivated vaccine rather than an attenuated one [49].

Today, two further virus infections have been eradicated globally through immunization: polio type 2 in 1999 [50] and rinderpest of cattle in 2011 [51]. Thanks to two different vaccines developed in the 1950s and 1960s, the killed vaccine by Jonas Salk and the live attenuated one by Albert Sabin, we are close to eradicating polio [50]. However, an anti-vaccine scare by a radical mullah in Nigeria [52] set back the eradication in West Africa by 10 years and allowed the same strain of polio virus to migrate to Indonesia, probably via the Haj. The use of ‘vaccinators' as a US cover for tracking down Osama bin Laden in Pakistan [53] has also hindered the polio endgame, including the tragic assassination of genuine public health vaccine workers in Pakistan in 2012 and again in 2014.

Vaccines are considered to be the most cost-effective public health interventions and it is estimated that between 2 and 3 million children in developing countries are spared from death every year [54]. In the 1990s, six vaccines were included in routine paediatric immunization programmes in many countries, and that number has increased to sixteen today. But we recognize that the full impact of vaccination has not been achieved, and major efforts are being made to develop vaccines against other diseases [55,56]. Equally important is the ongoing effort to make those vaccines available to all people of need around the world [54]. However, the development of preventive vaccines against the three largest infectious killer diseases (HIV/AIDS, malaria and tuberculosis) remains a challenge that will require a committed and continued effort towards the development of new scientific paradigms [57,58].

Since Jenner, the development of vaccines has been based on the re-creation of the protective immunity that results after natural infections. Novel vaccines against persistent infections may have to be ‘better than nature’, inducing the unnatural immune responses that nature has not learned to produce [56]. The power of vaccines is also being applied not only to prevent but also to treat diseases, such as cancer [59] and AIDS [60]. Moreover, vaccination is being explored as a measure to prevent non-infectious conditions, such as heroin and tobacco addictions [61]. The legacy of Jenner has extended beyond his wildest dreams!

## 7. Ethical considerations

A discussion on variolation and vaccination would not be complete without some comments on ethics. Every medical intervention implies the need for a risk–benefit assessment and the prevention of smallpox provides a good example. Although vilified by some, variolation could be justified on the terms of a more damaging alternative, namely to suffer the disease. Despite the inherent value of variolation, modern vaccinologists often consider variolation only as a procedure that facilitated the introduction of vaccination. However, variolation and vaccination coexisted in the UK until at least 1840, when variolation was outlawed by the Vaccination Act of that year. More controversial at that time was the Vaccination Act of 1853 that instituted compulsory vaccination. That decision led to movements that opposed vaccination, which on several occasions resulted in violent demonstrations [62]. Alfred Russel Wallace, who postulated evolution by natural selection independently of Charles Darwin, was deeply opposed to compulsory vaccination [63]. Eventually, in 1898, a new UK law was passed which permitted conscientious objectors [64].

Early developments in testing immunization procedures may appear high-handed by modern standards, such as the variolation of prisoners and of orphan children described in Sloane's paper. The ‘experiment’ conducted in 1796 by Jenner, by vaccinating and then variolating an 8 year old child may seem at first sight unethical today. However, children stood to benefit the most from vaccination and the challenge with smallpox ‘material’ (variolation) was in fact the ‘gold’ standard of smallpox prevention at that time [65].

Nonetheless, there continues to be a dilemma between the huge benefit of herd immunity for the population at large and the occasional deleterious side effect of vaccination in the rare individual, a matter of concern and liability for the pharmaceutical companies that produce vaccines. Moreover, there is still a sizeable anti-vaccination movement today [62]. The 0.3% mortality from variolation in Sloane's time was considered to be worth the risk, and the seriously adverse side effects of modern vaccines are far fewer; yet we recently witnessed how the entirely spurious scare of autism resulting from measles, mumps and rubella (MMR) combined vaccine led so many parents to avoid protecting their children. Thus, we applaud Sloane's sentiment [1] that it is a matter of wonder ‘that this operation which seems so plainly for the public good, should, through dread of the distempers being inculcated with it, and other unreasonable prejudices, be stopped from procuring it’.

## Author profiles

**Robin Weiss** is Emeritus Professor of Viral Oncology at University College London (UCL). He studied at UCL and worked in India, Czechoslovakia and USA. He was Director of the Institute of Cancer Research, London, 1980–1990. He has conducted research on HIV, oncogenic retroviruses and herpesviruses linked to lymphoma and Kaposi's sarcoma. Weiss is noted for discovering ‘fossil’ retroviral genomes in normal host DNA and that CD4 is the HIV receptor. He has spent much effort on HIV vaccine research, recently using single-chain llama antibodies, but regards himself as a ‘long-term non-progressor’ in this field. He delivered the 2014 Edward Jenner Lecture at St George's Hospital Medical School where Jenner trained. Weiss was President of the Society for General Microbiology, 2006–2009, and he currently chairs the Scientific Advisory Committee of the International AIDS Vaccine Initiative. He was elected a Fellow of the Royal Society in 1997 and a Foreign Associate of the US National Academy of Sciences in 2013.

**José Esparza** recently retired from the Bill & Melinda Gates Foundation where he worked for 10 years, serving as Senior Advisor on HIV Vaccines. Currently, he is an Adjunct Professor at the Institute of Human Virology (IHV), University of Maryland School of Medicine, where he also serves as Senior Advisor to the Global Virus Network (GVN). He also serves as a consultant in vaccinology to several companies and organizations. From 1986 to 2004, José was with the World Health Organization (WHO) and the Joint United Nations Programme on AIDS (UNAIDS) in Geneva, Switzerland, where he promoted the international development and testing of vaccines against HIV/AIDS. From 1974 to 1985, he worked at the Venezuelan Institute of Scientific Research (IVIC), where he became Professor of Virology, Head of the Laboratory of Biology of Viruses, and Chairman of the Center of Microbiology and Cell Biology. At that time he made numerous contributions in the area of rotavirology. In 1968, Esparza received his MD degree in Venezuela and in 1974 a PhD in Virology and Cell Biology from Baylor College of Medicine in Houston, Texas. He has published over 180 scientific articles, mostly in human virology, vaccinology and HIV/AIDS. He also has a major interest in the history of medicine, especially of infectious diseases and vaccination. He is member of the National Academy of Medicine in Venezuela, and serves in numerous advisory committees around the world. He has received numerous awards, including the 2013 Lifetime Achievement Award of the Institute of Human Virology, the 2013 Distinguished Alumnus Award of Baylor College of Medicine and the 2014 My Hero Award from AID for AIDS in New York. José currently lives in Reston, Virginia.
